# Activity and biophysical inhibition resistance of a novel synthetic lung surfactant containing Super-Mini-B DATK peptide

**DOI:** 10.7717/peerj.1528

**Published:** 2016-01-05

**Authors:** Robert H. Notter, Zhengdong Wang, Frans J. Walther

**Affiliations:** 1 Department of Pediatrics, University of Rochester, Rochester, NY, United States; 2 Department of Pediatrics, David Geffen School of Medicine, University of California, Los Angeles, CA, United States

**Keywords:** Acute respiratory distress syndrome (ARDS), Acute lung injury (ALI), Synthetic lung surfactant, Super Mini-B, Synthetic surfactant peptides, Neonatal respiratory distress syndrome (NRDS), SP-B peptide mimics, Super Mini-B DATK

## Abstract

**Background/objectives.** This study examines the surface activity, resistance to biophysical inhibition, and pulmonary efficacy of a synthetic lung surfactant containing glycerophospholipids combined with Super Mini-B (S-MB) DATK, a novel and stable molecular mimic of lung surfactant protein (SP)-B. The objective of the work is to test whether S-MB DATK synthetic surfactant has favorable biophysical and physiological activity for future use in treating surfactant deficiency or dysfunction in lung disease or injury.

**Methods.** The structure of S-MB DATK peptide was analyzed by homology modeling and by FTIR spectroscopy. The *in vitro* surface activity and inhibition resistance of synthetic S-MB DATK surfactant was assessed in the presence and absence of albumin, lysophosphatidylcholine (lyso-PC), and free fatty acids (palmitoleic and oleic acid). Adsorption and dynamic surface tension lowering were measured with a stirred subphase dish apparatus and a pulsating bubble surfactometer (20 cycles/min, 50% area compression, 37 °C). *In vivo* pulmonary activity of S-MB DATK surfactant was measured in ventilated rabbits with surfactant deficiency/dysfunction induced by repeated lung lavages that resulted in arterial PO_2_ values <100 mmHg.

**Results.** S-MB DATK surfactant had very high surface activity in all assessments. The preparation adsorbed rapidly to surface pressures of 46–48 mN/m at 37 °C (low equilibrium surface tensions of 22–24 mN/m), and reduced surface tension to <1 mN/m under dynamic compression on the pulsating bubble surfactometer. S-MB DATK surfactant showed a significant ability to resist inhibition by serum albumin, C16:0 lyso-PC, and free fatty acids, but surfactant inhibition was mitigated by increasing surfactant concentration. S-MB DATK synthetic surfactant quickly improved arterial oxygenation and lung compliance after intratracheal instillation to ventilated rabbits with severe surfactant deficiency.

**Conclusions.** S-MB DATK is an active mimic of native SP-B. Synthetic surfactants containing S-MB DATK (or related peptides) combined with lipids appear to have significant future potential for treating clinical states of surfactant deficiency or dysfunction, such as neonatal and acute respiratory distress syndromes.

## Introduction

When endogenous lung surfactant is deficient or becomes dysfunctional in humans, it can be replaced by exogenous surface-active substitutes. Therapy with current animal-derived exogenous surfactant drugs has proven to be life-saving in preventing and treating the neonatal respiratory distress syndrome (NRDS) in preterm infants, and on-going research is studying the feasibility of efficaciously extending surfactant-based therapies to pediatric and adult patients with clinical acute lung injury (ALI) or the acute respiratory distress syndrome (ARDS) (e.g., Raghavendran, Willson & Notter, 2011; Willson & Notter, 2011). Developing effective surfactant therapy for ALI/ARDS is particularly challenging, and requires the use of exogenous surfactants having maximal surface and pulmonary activity, plus the ability to resist inhibition from endogenous substances present in injured lungs in edema fluid or in association with the inflammatory response. There is significant interest in developing active synthetic lung surfactant drugs to help improve and optimize therapies of this kind.

Synthetic lung surfactants have a number of important advantages over current animal-derived surfactants as pharmaceutical products for treating NRDS and ALI/ARDS (Walther et al., 2007; Walther et al., 2010; Willson & Notter, 2011). In research on synthetic surfactant development, particular emphasis has been placed on designing peptide mimics of surfactant protein (SP)-B because of its powerful functional biophysical activity in interacting with lipids in endogenous pulmonary surfactant (Yu & Possmayer, 1988; Oosterlaken-Dijksterhuis et al., 1991a; Oosterlaken-Dijksterhuis et al., 1991b; Oosterlaken-Dijksterhuis et al., 1992; Seeger, Günther & Thede, 1992; Wang et al., 1996; Wang et al., 2002; Notter, 2000; Notter et al., 2002). In prior reports, we have detailed the molecular design, synthesis and activity of Mini-B (MB) and Super Mini-B (S-MB) peptides, two active mimics of native SP-B (Waring et al., 2005; Walther et al., 2007; Walther et al., 2010). These two active peptides (34 and 41 residues, respectively) incorporate active amphipathic helices and the “saposin fold” (Bruhn, 2005) of human SP-B (i.e., helix—turn—helix), and S-MB additionally includes the functionally important N-terminal insertion sequence of the native apoprotein. Here, we report the synthesis and high *in vitro* and *in vivo* activity of a novel SP-B peptide mimic, S-MB DATK, characterized by an added important designer-loop stabilizing substitution in the sequence of S-MB to increase molecular stability and improve the ease of synthesis and folding (Notter et al., 2012; Walther et al., 2013). The major focus of this study is on documenting the high surface activity and biophysical inhibition resistance of S-MB DATK synthetic surfactant, as well as its promising pulmonary activity in a rabbit model relevant for NRDS and ALI/ARDS.

Characterizations of surface activity examine both adsorption and dynamic surface tension lowering as physiologically-important interfacial properties, and inhibitor substances studied include serum albumin, lyso-PC and unsaturated free fatty acids. *In vivo* studies assess changes in lung function and compliance following the intratracheal instillation of S-MB DATK synthetic surfactant to mechanically-ventilated rabbits with surfactant deficiency/dysfunction induced by repeated lung lavage.

## Materials and Methods

### Super Mini-B DATK synthesis

S-MB DATK peptide (41 residues, amino acid sequence FPIPLPYCWLCRALIKRIQA- MIDATKRMLPQLVCRLVLRCS) was synthesized employing the same general protocol developed earlier for the MB and S-MB peptides (Waring et al., 2005; Walther et al., 2007; Walther et al., 2010; Walther et al., 2013; Notter et al., 2012). In brief, synthesis was done on a Symphony Multiple Peptide Synthesizer (Protein Technologies, Tucson, AZ) using a standard protocol on a H-Ser(OtBu)-HMPB NovaPEG resin (EMD Millipore, Billerica, MA, USA). All residues were double coupled to the resin to insure optimal yield at a 0.25 mmole scale. Crude S-MB DATK was cleaved from the resin using a cleavage-deprotection mixture of 0.75:0.25:0.5:0.5:10 (v:v) phenol:thioanisole:ethanedithiol:water:trifluoracetic acid (Applied Biosystems, 1990). The crude peptide was purified (better than 95%) by preparative HPLC using a VYDAC diphenyl or C8 (1″ by 12″ width by length) column at 20 mL/min. S-MB DATK was eluted from the column with a 0–100% (water to acetonitrile with 0.1% trifluoracetic acid as an ion pairing agent added to both aqueous and organic phases) linear gradient in one hour. Because of the enhanced peptide molecular stability imparted by the designer-loop DATK substitution in S-MB DATK, substantial treatment to further enhance folding/oxidation was not required. The purified peptide product eluted from the VYDAC column was freeze-dried from 10 mM HCl to remove residual trifluoracetic acid, desalted by dialysis, re-lyophilized, and the mass was confirmed by Maldi Time Of Flight mass spectrometry.

### Synthetic surfactant phospholipids

Synthetic phospholipids used in this study were dipalmitoyl phosphatidylcholine (DPPC), palmitoyl-oleoyl phosphatidylcholine (POPC), and palmitoyl-oleoyl phosphatidylglycerol (POPG). All phospholipids were obtained from Avanti Polar Lipids (Alabaster, AL, USA). Compounds were >99% pure as supplied and gave single spots on thin-layer chromatography with solvent system C of Touchstone, Chen & Beaver (1980).

### Synthetic surfactant mixture formulation

Synthetic surfactant mixtures were formulated to contain 5:3:2 (mole ratio) DPPC:POPC:POPG plus 3% by weight S-MB DATK peptide as follows. For surface activity studies, an aliquot of S-MB DATK peptide in trifluoroethanol was added to phospholipids in chloroform at the desired final composition ratio, and the organic solvents were evaporated under nitrogen. The resultant dry mixtures were stored overnight under house vacuum to remove residual solvent, and then dispersed in 0.15 M NaCl (pH 7.0) by hand vortexing and heating at 65 °C intermittently for about 30 min. The dispersion was stored at 5 °C for at least 12 h prior to testing. At the time of testing, surfactant was warmed to room temperature and again mechanically vortexed by hand a few times prior to use. Surfactant formulation methods for *in vivo* activity studies were similar, but incorporated lyophilization and rotary evaporation to prepare a single large batch of dispersed synthetic surfactant.

### Inhibitor compounds

Bovine serum albumin (BSA), synthetic C16:0 Lysophosphatidylcholine (lyso-PC), palmitoleic (C16:1) acid (PA), and oleic (C18:1) acid (OA) were purchased from the Sigma Chemical Co. (St. Louis, MO, USA) for use in inhibitor studies. BSA was essentially free of fatty acid and was prepared from fraction V albumin. Lyso-PC, PA, and OA were reagent grade (99% pure). For surfactant inhibition experiments, an inhibitor compound dispersed in buffer (or ethanol in the case of the PA and OA) was added at known concentration to a surfactant dispersion, followed by gentle vortexing. Aliquots of surfactant/inhibitor mixtures were examined in adsorption or pulsating bubble studies within 2 h of preparation.

### Molecular modeling and Fourier transform infrared (FTIR) spectroscopy to determine the secondary structure of S-MB DATK

The theoretical structure for the amino acid sequence of reduced S-MB DATK peptide was predicted using the homology-based structure software I-TASSER 3.0 (Zhang, 2008; Roy, Kucukural & Zhang, 2010) at http://zhanglab.ccmb.med.umich.edu/I-TASSER. The homology-modeled structure was rendered using PyMOL (TM) 1.7.4.1 (Schrodinger, LLC, Cambridge, MA, USA). Infrared spectra of S-MB DATK in synthetic surfactant lipid multilayers were measured at 37 °C with a Bruker Vector 22 FTIR spectrometer (Pike Technologies, Madison, WI, USA) fitted with a deuterium triglyceride sulfate (DTGS) detector. For FTIR studies, S-MB DATK was co-solvated with surfactant lipids (DPPC:POPC:POPG; molar ratio, 5:3:2) in trifluorethanol:chloroform, 1:1, v:v (lipid:peptide mole ratio of 10:1) and transferred onto an ATR crystal (Pike Technologies, Fitchburg, WI, USA). The organic solvent was removed by flowing nitrogen gas over the sample to produce a lipid-peptide multilayer film, which was then hydrated (≥35%) with deuterium vapor in nitrogen for 1 h prior to acquiring spectra (Gordon et al., 1996; Gordon et al., 2000; Yamaguchi et al., 2002). FTIR data were averaged over 256 scans at a gain of 4 and a resolution of 2, and final spectra for S-MB DATK in the lipid multilayer were obtained by subtracting the lipid spectrum with D_2_O from that of peptide in lipid with D_2_O hydration. Relative amounts of *α*-helix, *β*-turn, *β*-sheet, or random (disordered) structures in either peptide self-films or lipid-peptide films were estimated using Fourier self-deconvolution (GRAMS/AI8, version 8.0, Themo Electron Corporation, Waltham, MA, USA) and area of component peaks calculated using curve-fitting software (Igor Pro, version 1.6, Wavemetrics, Lake Oswego, OR) (Kauppine et al., 1981). FTIR frequency limits were: *α*-helix (1,662–1,645 cm^−1^), *β*-sheet (1,637–1,613 cm^−1^ and 1,710–1,682 cm^−1^), turn/bend (1,682–1,662 cm^−1^), and disordered or random (1,650–1,637 cm^−1^) (Byler & Susi, 1986).

### Pulsating bubble methods

A pulsating bubble surfactometer (Electronetics, General Transco, St Petersburg, FL, USA) based on the original design of Enhorning (Enhorning, 1977) was used to measure the dynamic surface tension lowering of synthetic surfactant mixtures in the presence and absence of inhibitors. Measurements with this instrument reflect the combined effects of adsorption and dynamic film compression, and were performed as a function of surfactant and inhibitor concentrations at 37 °C. A small air bubble, communicating with ambient air, was formed in an aqueous dispersion of synthetic S-MB DATK surfactant (with or without inhibitors) held in a small plastic sample chamber. This bubble was then pulsated between maximum and minimum radii of 0.55 and 0.4 mm (50% area compression). Surface tension at minimum bubble radius (minimum surface tension) was calculated as a function of time from the Laplace equation and the measured pressure drop across the bubble interface (Enhorning, 1977; Hall et al., 1993; Notter, 2000).

### Adsorption methods

Adsorption experiments were done at 37 °C in a Teflon dish with a 35 mL subphase (0.15 M NaCl, pH 7.0) stirred to minimize diffusion resistance (Notter, Taubold & Finkelstein, 1983; Notter, 2000). At time zero, a bolus of S-MB DATK surfactant containing 2.5 mg phospholipid in 5 mL of 0.15 M NaCl with pH 7.0 was injected into the stirred subphase, and adsorption surface pressure (surface tension lowering below that of the pure subphase) was measured as a function of time by the force on a partially submerged, sandblasted platinum Wilhelmy slide (Notter et al., 1982; Notter, Taubold & Finkelstein, 1983; Notter, 2000). Final adsorption subphase concentration of surfactant was uniform at 0.0625 mg phospholipid/mL (doubled for high surfactant concentration studies).

### *In vivo* surfactant activity studies in lavaged, surfactant-deficient rabbits

The animal study was reviewed and approved by the Institutional Animal Care and Use Committee of the Los Angeles Biomedical Research Institute at Harbor-UCLA Medical Center (Research Project # 12958). All procedures and anesthesia were in accordance with the American Veterinary Medical Association (AMVA) Guidelines. Young adult New Zealand white rabbits (weight 1.0–1.3 kg) received anesthesia with 50 mg/kg of ketamine and 5 mg/kg of acepromazine intramuscularly prior to placement of a venous line via a marginal ear vein. After intravenous (IV) administration of 1 mg/kg of diazepam and 0.2 mg/kg of propofol, a small skin incision in the anterior neck was made to allow placement of an endotracheal tube and carotid arterial line. Muscle paralysis was induced with IV pancuronium (0.1 mg/kg, with repeat doses hourly for maintenance) and rabbits were ventilated with a Harvard volume-controlled animal ventilator (tidal volume 7.5 mL/kg, positive end-expiratory pressure 3 cm H_2_O, a 1:2 ratio of inspiration:expiration, 100% oxygen, and a respiratory rate to maintain an arterial PCO_2_ at ∾40 mmHg). Anesthesia was maintained throughout the study by continuous IV administration of 3 mg/kg/h of propofol, with supplemental doses of IV diazepam (1 mg/kg) as needed. Heart rate, arterial blood pressures, and rectal temperature were monitored continuously, and ventilator airway flow, pressures, and tidal volume were recorded with a pneumotachograph connected to the endotracheal tube and a pneumotach system. Edema fluid appearing in the trachea was removed by suctioning as needed, and maintenance fluid was provided by infusion of Lactated Ringer’s solution at a steady rate of 10 mL/kg/h. After initial stabilization on the ventilator, saline lavage was performed with repeated intratracheal instillation and removal of 30 mL of normal saline until arterial PO_2_ dropped below 100 mmHg (average 3 lavages). When PaO_2_ was stable at less than 100 mmHg, S-MB DATK synthetic surfactant or lipid-only control was instilled into the trachea at a dose of 100 mg/kg body weight and a concentration of 35 mg/mL. Pulmonary gas exchange (arterial pH, PCO_2_ and PO_2_) and ventilator dynamic compliance was determined every 15 min over a 2-hour post-instillation study period as primary endpoints. Dynamic compliance was calculated by dividing ventilator tidal volume/kg body weight by changes in airway pressure (peak inspiratory pressure minus positive end-expiratory pressure) (mL/kg/cm H_2_O). Animals were sacrificed at the end of study with an overdose of IV pentobarbital.

### Statistical analysis

All data are expressed as mean ±SEM. Statistical analyses used Student’s *t*-test for comparisons of discrete data points, and functional data were analyzed by one-way analysis of variance (ANOVA) with Scheffe’s *post hoc* analysis to adjust for multiple comparisons. Differences were considered statistically significant if the *P* value was <0.05.

## Results

**Figure 1:**
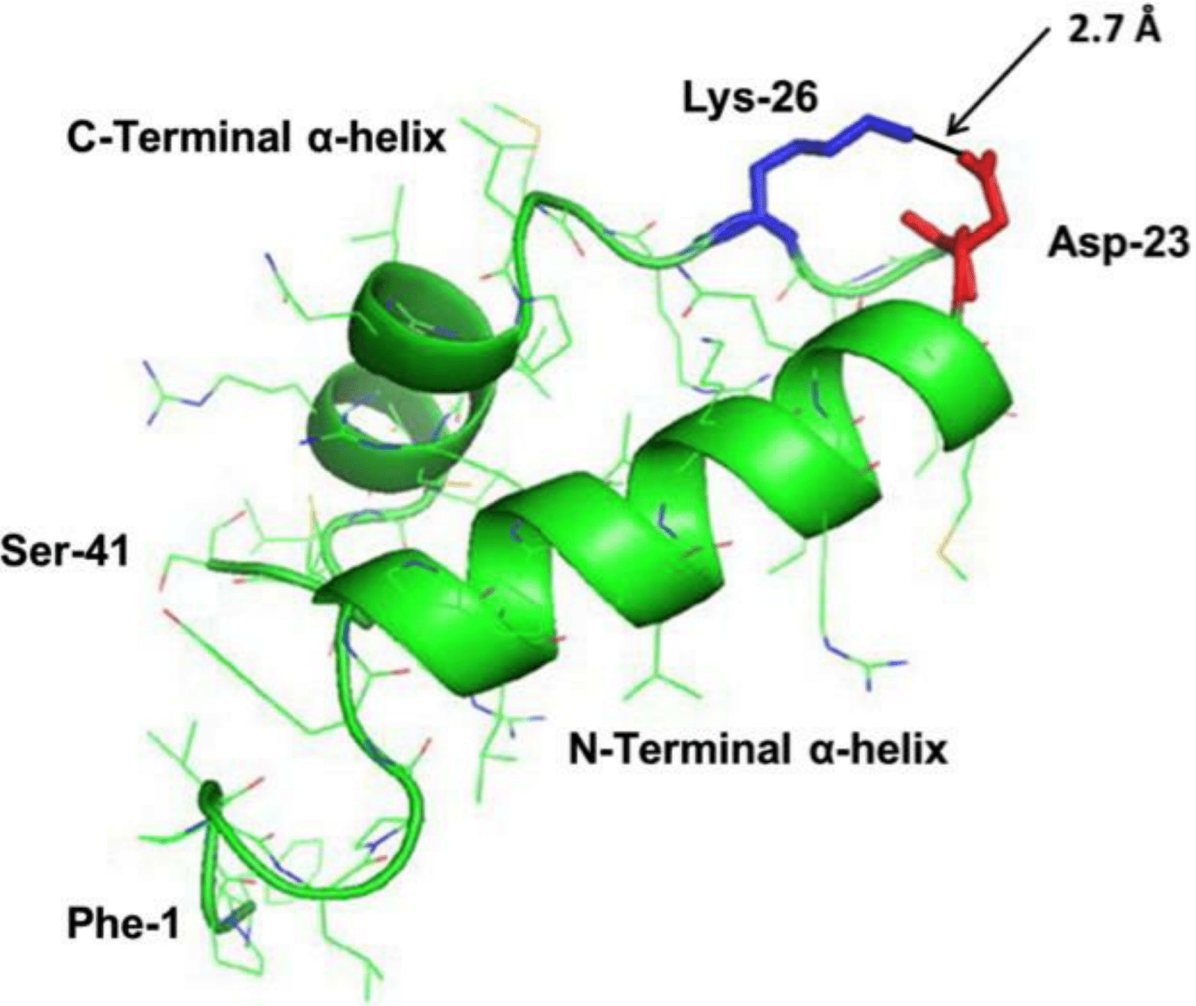
Homology-modeled secondary structure of reduced S-MB DATK peptide. The predicted homology-modeled structure from the amino acid sequence of S-MB DATK peptide was calculated via I-TASSER 3.0 (Zhang, 2008; Roy, Kucukural & Zhang, 2010) (http://zhanglab.ccmb.med.umich.edu/I-TASSER) and rendered using PyMOL (TM). Helical residues are highlighted in green ribbon, disordered and loop-turn segments are represented as green tubes, and the ion-pair that stabilizes the turn is rendered in red (Asp^−^-23) and blue (Lys^+^-26). The characteristic “saposin fold” of the reduced S-MB DATK encompasses the N-terminal *α*-helix (residues 8–21), the loop-turn (residues 22–29) and C-terminal *α*-helix (residues 30–37), while the additional N-terminal insertion sequence includes residues 1–7 (see text).

**Figure 2:**
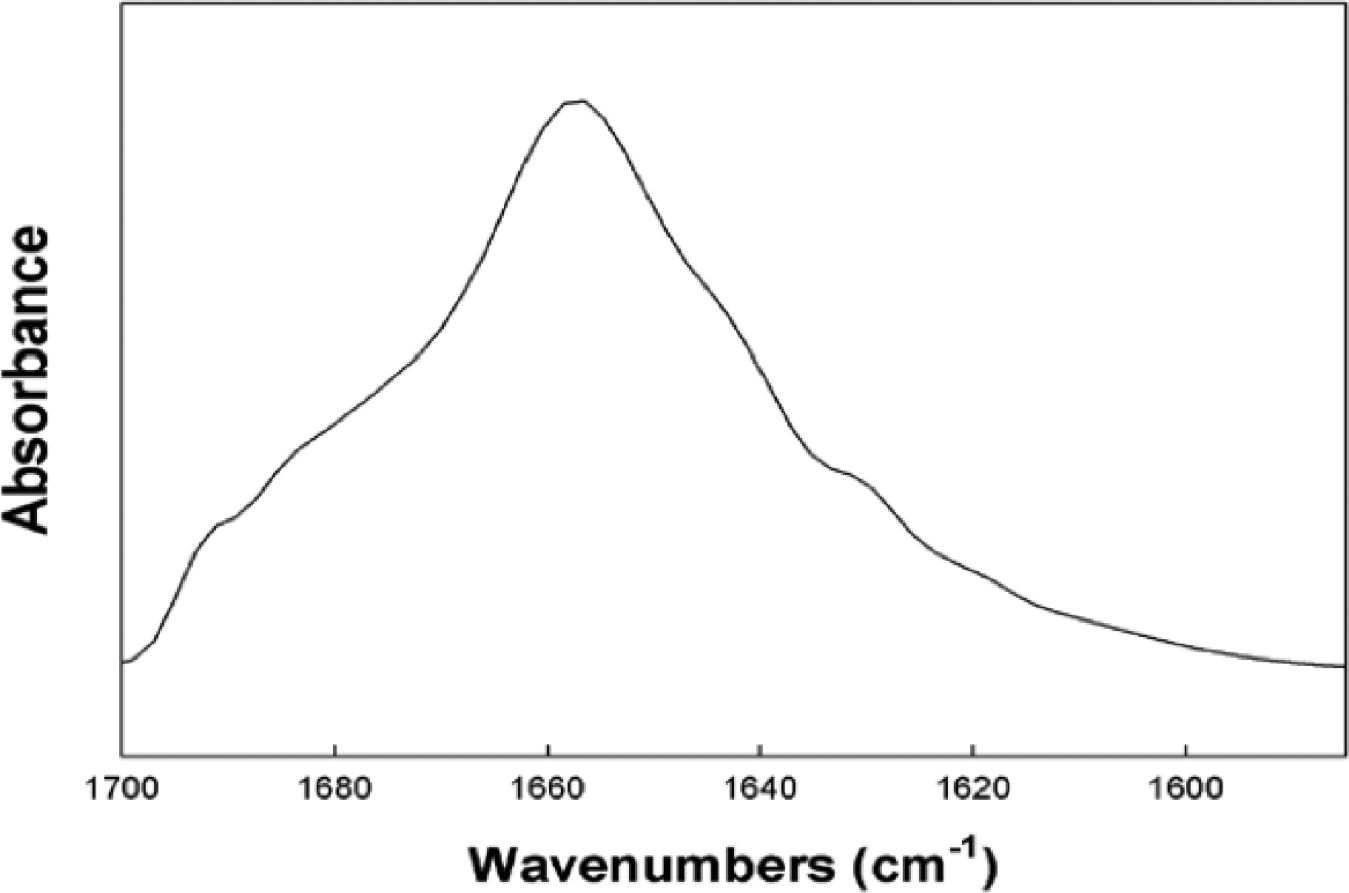
Representative FTIR spectrum of reduced S-MB DATK in surfactant lipids. FTIR spectra were measured for reduced S-MB DATK in multilayer films with synthetic surfactant lipids (5:3:2 DPPC:POPC:POPG) (Methods). The peptide to lipid mole ratio in the mixed film was 1:10. The representative FTIR spectrum shown is consistent with the primary overall helix-turn-helix motif of homology-modeled S-MB DATK in Fig. 1. Proportions of specific secondary structure determined for reduced S-MB DATK by Fourier self-deconvolution from the Amide I band of the spectra are given in Table 1. See text for details.

Predictive homology modeling of S-MB DATK structure is shown in Fig. 1 based on analysis of its amino acid sequence using an iterative threading-assembly algorithm approach with I-TASSER 3.0 (Zhang, 2008; Roy, Kucukural & Zhang, 2010). Homology-modeled S-MB DATK peptide had an overall helix-turn-helix saposin-related structural motif, as reported previously for the related MB and S-MB peptides (Waring et al., 2005; Walther et al., 2010). To gain further direct information on the molecular structure of S-MB DATK in lipid environments, FTIR measurements assessed peptide secondary structure in multilayers of 5:3:2 DPPC:POPC:POPG (Fig. 2). The FTIR spectrum of reduced S-MB DATK in synthetic lipids in Fig. 2 is very similar to those reported previously for oxidized MB and S-MB in their fully disulfide-stabilized state (Walther et al., 2010). Deconvolution analysis of FTIR spectra for reduced S-MB DATK showed that the peptide had a predominant alpha helical conformation in synthetic surfactant lipids, together with a significant proportion of stable bend-loop beta structure and low amounts of non-specific beta structural formation (Table 1).

**Table 1:** FTIR-derived proportions of secondary structures of reduced S-MB DATK peptide in multilayers of surfactant lipids. The synthetic peptide was mixed in a 1:10 proportion to surfactant lipids and studied by ATR-FTIR spectroscopy. Relative amounts of *α*-helix, *β*-turn (loop-turn), *β*-sheet, or random (disordered) structures in peptide self-films or lipid-peptide films were calculated by Fourier self-deconvolution of the amide I band of the spectra (see Fig. 2 for representative spectrum and Methods for details). See Methods section for FTIR experimental details.

	% conformation			
	*α*-helix	*β*-sheet	Loop-turn	Disordered
S-MB DATK:lipids	41.98	12.83	27.52	17.67

The high *in vitro* adsorption and dynamic surface activity of 5:3:2 DPPC:POPC:POPG +3% S-MB DATK surfactant in the absence of inhibitor substances is summarized in Table 2. Synthetic S-MB DATK surfactant rapidly adsorbed to the equilibrium spreading limit of phospholipids (i.e., surface pressures of the order of 46–48 mN/m at 37 °C, equivalent to equilibrium surface tensions of 22–24 mN/m). This rapid adsorption was found despite the very low concentration of 0.0625 mg surfactant phospholipid/mL used in the studies (Table 2A). Synthetic S-MB DATK surfactant also reached very low minimum surface tensions <1 mN/m during dynamic compression on a pulsating bubble apparatus at physical conditions reflective of normal respiration (20 cycles/min, 50% area compression, 37 °C) (Table 2B).

**Table 2:** Surface activity of a synthetic lung surfactant containing S-MB DATK peptide combined at 3% (by wt) with glycerophospholipids (5:3:2 DPPC:POPC:POPG). Adsorption results are expressed in terms of surface pressure, defined as the amount of surface tension lowering below that of the pure subphase at the experimental temperature (70.3 mN/m for water at 37 ± 0.5 °C). Higher surface pressure is equivalent to lower surface tension. Final surfactant concentration for adsorption was 0.0625 mg phospholipid/mL (2.5 mg surfactant phospholipid in 40 mL of final subphase). Dynamic surface tension was measured with a pulsating bubble surfactometer (37 °C, 50% area compression, 20 cycles/min) at a surfactant concentration of 5 mg/mL in normal saline at pH 7.0. Data are mean ± SEM for *n* = 4–5.

(A) Adsorption surface pressure (mN/m) vs. time following injection beneath a stirred subphase						
0 (min)	1	2	5	10	15	20
0.0 ± 0.0	45.3 ± 1.5	46.0 ± 1.7	47.3 ± 1.2	47.5 ± 0.9	47.5 ± 0.7	47.7 ± 0.6

(B) Dynamic surface tension (mN/m) vs. time during pulsation on a bubble surfactometer							
0.25 (min)	0.5	1	2	5	10	15	20
22.9 ± 0.8	21.7 ± 0.8	20.0 ± 0.3	17.9 ± 0.7	14.8 ± 1.9	10.9 ± 2.2	2.9 ± 1.8	0.0 ± 0.0

**Figure 3:**
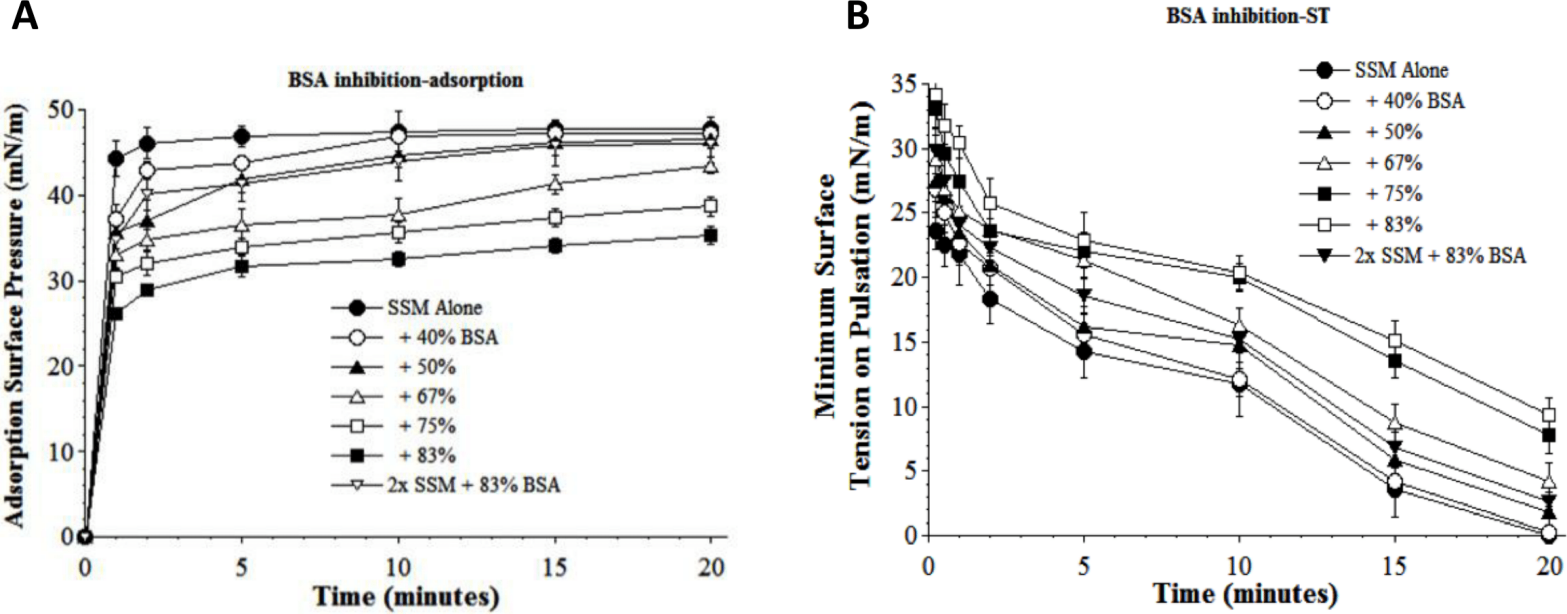
Inhibitory effect of bovine serum albumin (BSA) on the adsorption (A) and dynamic surface tension lowering (B) of synthetic surfactant mixtures. (A) Surface pressure (amount by which surface tension is lowered below the pure subphase value of 70 mN/m at 37 °C) is plotted as a function of time during adsorption. Synthetic surfactant mixtures with or without BSA were injected at time zero beneath the surface of a well-stirred buffered subphase. Final adsorption subphase concentration of synthetic surfactant mixtures was 0.0625 mg/mL (doubled in high concentration surfactant case). Data are mean ± SEM, *n* = 3–5. All adsorption curves with 50–83% albumin present are significantly different from SSM alone, and curve for 2x SSM concentration with 83% albumin is significantly improved compared to the equivalent low surfactant concentration curve at the same albumin level (*P* < 0.05 or less by one-way ANOVA). (B) Data are minimum surface tension as a function of time for dispersions of surfactant plus inhibitors in a pulsating bubble surfactometer (20 cycles/min at 37 °C). Surfactant phospholipid concentration was 5.0 mg/mL (doubled in high concentration surfactant case). Data are mean ± SEM, *n* = 3–5. All dynamic surface tension lowering curves with albumin present at 50–83% are significantly different from SSM alone, and curve for 2x SSM concentration with 83% albumin is significantly improved compared to the equivalent low surfactant concentration curve at the same albumin level (*P* < 0.05 or less by one-way ANOVA). See Methods and text for details.

Additional *in vitro* surface activity studies examined the ability of synthetic S-MB DATK surfactant to resist biophysical inhibition by substances that can be present in the lung alveoli or interstitium during inflammatory lung injury. The first inhibitor studied was the plasma protein albumin, a prominent component of high molecular weight pulmonary edema. Bovine serum albumin (BSA) induced a concentration-dependent inhibition of the adsorption and dynamic surface tension lowering of S-MB DATK surfactant (5:3:2 DPPC:POPC:POPG + 3% S-MB DATK, abbreviated as SSM in Fig. 3). The inhibitory effects of BSA on adsorption and dynamic surface tension lowering were small at a concentration of 40% by weight, but became progressively more pronounced as albumin levels were increased by weight relative to surfactant. However, albumin-induced reductions of adsorption and dynamic surface activity were significantly mitigated by increasing the concentration of surfactant at the highest plasma protein level studied (83%, Fig. 3).

**Figure 4:**
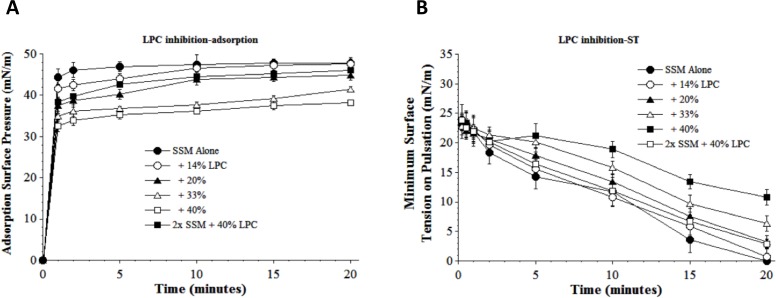
Inhibitory effect of Lyso-PC (LPC) on the adsorption (A) and dynamic surface tension lowering (B) of synthetic surfactant mixtures. (A) Surface pressure (amount by which surface tension is lowered below the pure subphase value of 70 mN/m at 37 °C) is plotted as a function of time during adsorption. Synthetic surfactant mixtures with or without LPC were injected at time zero beneath the surface of a well-stirred buffered subphase. Final adsorption subphase concentration of synthetic surfactant mixtures was 0.0625 mg/mL (doubled in high concentration surfactant case). Data are mean ± SEM, *n* = 3–5. All adsorption curves with 20–40% LPC present are significantly different from SSM alone, and curve for 2x SSM concentration with 40% LPC is significantly improved compared to the equivalent low surfactant concentration curve at the same LPC level (*P* < 0.05 or less by one-way ANOVA). (B) Data are minimum surface tension as a function of time for dispersions of surfactant plus inhibitors in a pulsating bubble surfactometer (20 cycles/min at 37 °C). Surfactant phospholipid concentration was 5.0 mg/mL (doubled in high concentration surfactant case). Data are mean ± SEM, *n* = 3–5. All dynamic surface tension lowering curves with LPC present at 20–40% are significantly different from SSM alone, and curve for 2x SSM concentration with 40% LPC is significantly improved compared to the equivalent low surfactant concentration curve at the same LPC level (*P* < 0.05 or less by one-way ANOVA). See Methods and text for details.

A second physiologically-relevant inhibitor substance studied was lyso-PC, which acts to compromise surface activity by a different biophysical mechanism than albumin (Wang & Notter, 1998; Holm, Wang & Notter, 1999; Notter, 2000). Lyso-PC can be present in injured lungs as a result of cell damage or endogenous phospholipase activity during the inflammatory response. Lyso-PC significantly decreased both the adsorption and dynamic surface tension lowering ability of S-MB DATK surfactant (SSM) in proportion to inhibitor concentration (Figs. 4A and 4B, respectively). However, even at a high lyso-PC concentration of 40%, detrimental effects on adsorption could be significantly mitigated by increasing SSM concentration (Fig. 4A). A similar mitigation of the inhibitory effect of 40% lyso-PC on dynamic surface tension lowering ability was also observed when surfactant concentration was increased (Fig. 4B).

**Figure 5:**
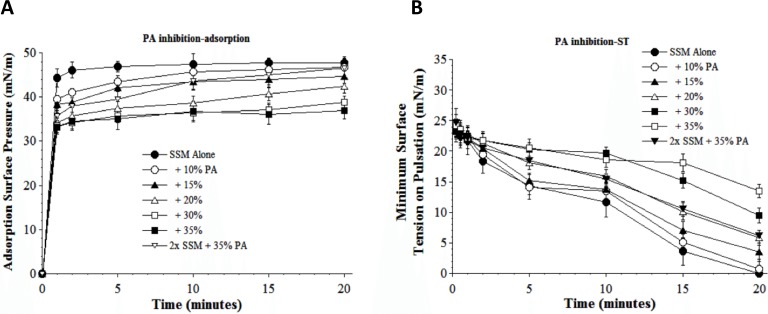
Inhibitory effect of palmitoleic acid (PA) on the adsorption (A) and dynamic surface tension lowering (B) of synthetic surfactant mixtures. (A) Surface pressure (amount by which surface tension is lowered below the pure subphase value of 70 mN/m at 37 °C) is plotted as a function of time during adsorption. Synthetic surfactant mixtures with or without PA were injected at time zero beneath the surface of a well-stirred buffered subphase. Final adsorption subphase concentration of synthetic surfactant mixtures was 0.0625 mg/mL (doubled in high concentration surfactant case). Data are mean ± SEM, *n* = 3–5. All adsorption curves with 10–35% PA present are significantly different from SSM alone, and curve for 2x SSM concentration with 35% PA is significantly improved compared to the equivalent low surfactant concentration curve at the same PA level (*P* < 0.05 or less by one-way ANOVA). (B) Data are minimum surface tension as a function of time for dispersions of surfactant plus inhibitors in a pulsating bubble surfactometer (20 cycles/min at 37 °C). Surfactant phospholipid concentration was 5.0 mg/mL (doubled in high concentration surfactant case). Data are mean ± SEM, *n* = 3–5. All dynamic surface tension lowering curves with PA present at 20–35% are significantly different from SSM alone, and curve for 2x SSM concentration with 35% PA is significantly improved compared to the equivalent low surfactant concentration curve at the same PA level (*P* < 0.05 or less by one-way ANOVA). See Methods and text for details.

**Figure 6:**
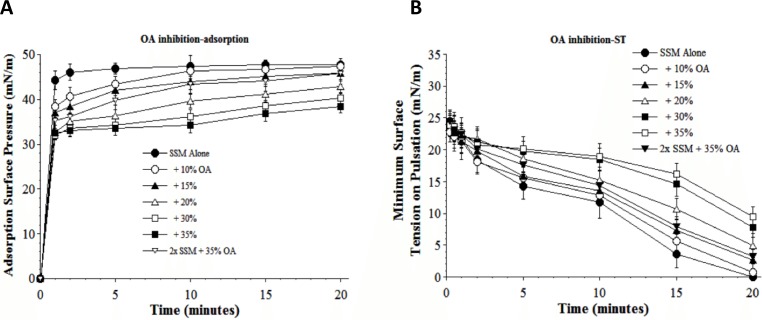
Inhibitory effect of oleic acid (OA) on the adsorption (A) and dynamic surface tension lowering (B) of synthetic surfactant mixtures. (A) Surface pressure (amount by which surface tension is lowered below the pure subphase value of 70 mN/m at 37 °C) is plotted as a function of time during adsorption. Synthetic surfactant mixtures with or without OA were injected at time zero beneath the surface of a well-stirred buffered subphase. Final adsorption subphase concentration of synthetic surfactant mixtures was 0.0625 mg/mL (doubled in high concentration surfactant case). Data are mean ± SEM, *n* = 3–5. All adsorption curves with 10–35% OA present are significantly different from SSM alone, and curve for 2x SSM concentration with 35% OA is significantly improved compared to the equivalent low surfactant concentration curve at the same OA level (*P* < 0.05 or less by one-way ANOVA). (B) Data are minimum surface tension as a function of time for dispersions of surfactant plus inhibitors in a pulsating bubble surfactometer (20 cycles/min at 37 °C). Surfactant phospholipid concentration was 5.0 mg/mL (doubled in high concentration surfactant case). Data are mean ± SEM, *n* = 3–5. All dynamic surface tension lowering curves with OA present at 20–35% are significantly different from SSM alone, and curve for 2x SSM concentration with 35% OA is significantly improved compared to the equivalent low surfactant concentration curve at the same OA level (*P* < 0.05 or less by one-way ANOVA). See Methods and text for details.

Results in Figs. 5 and 6 show the inhibitory effects on SSM adsorption and dynamic surface tension lowering from two fluid unsaturated free fatty acids (PA, C16:1 and OA, C18:1) that can also potentially be present in injured lungs as a result of cellular membrane damage and phospholipase activity. Both fatty acids decreased the adsorption of SSM in direct relation to inhibitor concentration (increasing inhibition as fatty acid concentration was raised from 10 to 35% by weight at low surfactant concentration) (Figs. 5A and 6A). The fatty acids also significantly impaired the ability of SSM to reach low surface tensions during dynamic cycling on a pulsating bubble apparatus (Figs. 5B and 6B). However, increasing the concentration of SSM reversed fatty acid-induced inhibition of both adsorption and dynamic surface activity even in the continued presence of maximum amounts of these inhibitors (Figs. 5 and 6).

**Figure 7:**
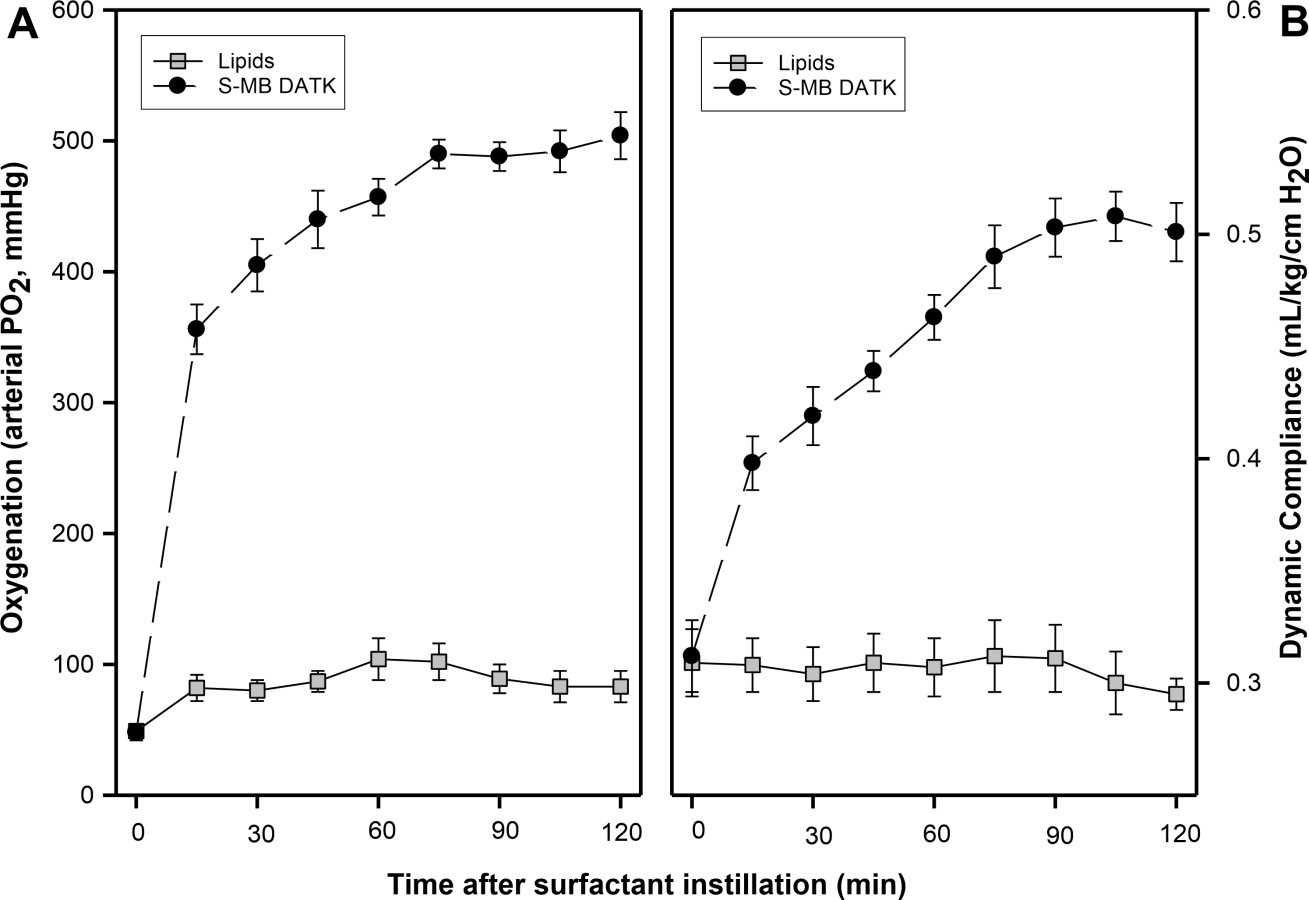
Effect of S-MB DATK synthetic surfactant on oxygenation and lung compliance in rats with surfactant-deficient ARDS. Synthetic surfactant or lipid-only control was instilled intratracheally at time zero into ventilated rats following *in vivo* lavage to induce clinical oxygenation criteria for ARDS. Arterial oxygenation (A) and dynamic lung compliance (B) are shown as a function of time following tracheal instillation of S-MB DATH surfactant or lipid-only control. Data are mean ± SEM for *n* = 3–5. Values for both oxygenation and dynamic compliance at all times greater than zero are significantly improved for surfactant-treated animals compared to controls (*P* < 0.001 by one-way ANOVA). See Methods and text for details.

An important final set of experiments directly examined the *in vivo* pulmonary activity of S-MB DATK synthetic surfactant when instilled intratracheally in ventilated rabbits with surfactant deficiency and impaired respiratory function induced by repeated saline lung lavages (Fig. 7). S-MB DATK surfactant was administered by intratracheal instillation after the arterial partial pressure of oxygenation was reduced to stable levels <100 mmHg, which is well below the threshold clinical criteria for ARDS (i.e., PaO_2_ < 200 mmHg when breathing 100% oxygen) (Bernard et al., 1994; Artigas et al., 1998). Rabbits receiving S-MB DATK surfactant had significantly improved arterial oxygenation over a 120 min period of study post-instillation compared to control rabbits instilled with surfactant lipids alone (Fig. 7). Lung compliance (ventilator lung compliance) was also significantly improved over the same period of post-instillation study in surfactant-treated rabbits compared to lipid-only controls (Fig. 7).

## Discussion

A major goal of this study was to progress towards the development of fully synthetic lung surfactants that have high surface and pulmonary activity as well as an ability to resist biophysical inhibition by endogenous substances present in the lungs during inflammatory injury. The synthetic surfactant studied contained the novel S-MB DATK peptide combined with synthetic glycerophospholipids (5:3:2 DPPC:POPC:POPG). This novel synthetic peptide contains key functional regions of active human SP-B including its N- and C-terminal amphipathic helices and N-terminal lipophilic insertion sequence, along with a specific designer-loop DATK substitution to further increase molecular stability and improve the ease of synthesis, folding and purification.

Structural homology modeling of S-MB DATK indicated the basic folding motif (i.e., helix-turn-helix) characteristic of saposin proteins (Bruhn, 2005). Predicted N- and C-terminal helices in reduced S-MB DATK spanned residues 8–21 and 30–37, respectively, with the nearly antiparallel *α*-helices interacting through hydrophobic interactions between side chains at the interface (Fig. 1). The helical topography for reduced S-MB DATK is similar to that of oxidized (i.e., disulfide-linked) Mini-B using 2D-NMR (PDB accession code: 2DWF) (Sarker et al., 2007) or ^13^C-FTIR spectroscopy (PDB: 1SSZ) (Waring et al., 2005). The predicted structure of reduced S-MB DATK is consistent with the formation of a strong, stabilizing neutral ion-pair (“salt-bridge” or “ion-lock”) between the Asp^−^-23 and Lys^+^-26 residues. The calculated 2.7 Å distance between the nearest Asp^−^-23 “O” and the Lys^+^-26 “N” in S-MB DATK peptide (Fig. 1) is sufficiently small to facilitate this kind of strong salt-bridge interaction. Stabilizing salt-bridges between Arg^+^ or Lys^+^ and Glu^−^ or Asp^−^ have previously been defined in several PDB-deposited structures having a close-range cutoff of ≤4.0 Å between the “N” of the cationic residue and the nearest “O” of the anionic residue (Kumar & Nussinov, 1999; Kumar & Nussinov, 2002; Kumar, Tsai & Nussinov, 2000). The incorporation of designed ion-locks to increase peptide molecular stability and activity has also been reported in recent studies by our group with a novel SP-C mimic (Walther et al., 2014b), and ion-locks have previously been used in other designer peptides as a ‘Molecular Velcro^®^’ to enhance molecular stability or activity (Huyghues-Despointes et al., 2006; Chapman et al., 2008; He et al., 2008).

FTIR analyses here (Fig. 2, Table 1) were also consistent with the presence of ion-lock (salt-bridge) formation in reduced S-MB DATK that stabilized its overall helix – turn – helix motif even in the absence of intra-peptide disulfide linkages. Major *α*-helical and *β*-turn components identified in the FTIR spectra of reduced S-MB DATK (Fig. 2; Table 1) were in agreement with those of the previously reported active oxidized SP-B mimics MB and S-MB (Waring et al., 2005; Walther et al., 2010; Walther et al., 2014a). FTIR spectral analysis of reduced S-MB DATK also showed low amounts of non-specific structures (Table 1), suggesting minimal peptide aggregation. Importantly, the stable *α*-helical and *β*-turn structures defined by FTIR in reduced S-MB DATK were achieved without the extensive post-synthesis modification and folding that is required for MB and S-MB peptides (Waring et al., 2005; Walther et al., 2007; Walther et al., 2010). These favorable molecular characteristics for reduced S-MB DATK translate to an enhanced ease of peptide production/purification, and also provide an improved potential to maintain a high stable activity in synthetic lung surfactant applications.

A series of interfacial biophysical studies showed that synthetic S-MB DATK surfactant containing phospholipids (5:3:2 DPPC:POPC:POPG) combined with 3% (by wt.) peptide had very high surface activity and inhibition resistance (Table 2, Figs. 3–6) In the absence of inhibitors, S-MB DATK synthetic surfactant adsorbed rapidly at very low concentration to reach very high surface pressures of 46–48 mN/m, values at or near the equilibrium spreading limit of fluid phospholipids (Chapman, 1973, Table 2). Synthetic S-MB DATK surfactant also reached very low minimum surface tensions of <1 mN/m during dynamic cycling at physical conditions relevant for normal human respiration (20 cycles/min, 50% area compression, 37 °C) (Table 2). The adsorption and dynamic surface activity of S-MB DATK surfactant was reduced by exposure to high concentrations of selected inhibitor substances that can be present in the lungs during inflammatory lung injury (Figs. 3–6). However, S-MB DATK surfactant showed significant resistance to the biophysical inhibitors by mitigating their detrimental effects on surface activity when surfactant concentration was raised.

Specific inhibitors of surfactant activity studied were serum albumin, lyso-PC, and unsaturated free fatty acids. Albumin was used as a representative plasma/blood protein known to be present in high molecular weight pulmonary edema in clinical ALI/ARDS (Bernard et al., 1994; Artigas et al., 1998). Mechanistically, large plasma proteins like albumin inhibit surface activity primarily by adsorbing to the air–water interface and hindering the entry of lung surfactant components into the surface film (Holm, Enhorning & Notter, 1988; Holm, Wang & Notter, 1999). Lyso-PC and unsaturated free fatty acids (PA and OA) were studied as additional physiologically-relevant inhibitors that can be present in injured or inflamed lungs (Hall et al., 1992; Holm, Wang & Notter, 1999). Lyso-PC and fluid free fatty acids act mechanistically to compromise surfactant activity not only by affecting adsorption, but also by directly mixing into the lipid-rich interfacial film to impair its ability to lower surface tension during dynamic compression (Hall et al., 1992; Holm, Wang & Notter, 1999). The ability of S-MB DATK synthetic surfactant to overcome biophysical inhibition by all four of the inhibitors studied (albumin, LPC, PA, and OA) despite their diverse mechanisms of action supports its potential to maintain high activity in states of inflammatory lung injury.

In addition to directly documenting the beneficial inhibition-resistance of S-MB DATK surfactant, another important aspect of study here was to directly define the *in vivo* effectiveness of this synthetic preparation when instilled intratracheally into animals with lung injury and surfactant deficiency relevant for the neonatal respiratory distress syndrome (NRDS) and ALI/ARDS. Consistent with its significant surface activity and inhibition resistance, S-MB DATK synthetic surfactant generated substantial improvements in acute lung function (arterial blood gases) and lung mechanics (dynamic lung compliance) following intratracheal administration to mechanically ventilated, lung lavaged rabbits that met oxygenation criteria for clinical ARDS (Fig. 7). Animal experiments were acute in nature, and examined surfactant-associated pulmonary variables over a 2 h period post-instillation. The partial pressure of arterial oxygen in rabbits ventilated with 100% oxygen was improved rapidly and significantly at 15 min post-instillation of S-MB DATK surfactant (356 ± 19 mmHg compared to 82 ± 10 mmHg in lipid-only controls) (Fig. 7). Arterial oxygenation further improved to a final value of 504 ± 18 mmHg in surfactant-treated animals at two hours post-instillation compared to only 83 ± 12 mmHg in controls. Calculated values of dynamic lung compliance showed a similar pattern of rapid and significant improvement by 15 min following instillation of synthetic S-MB DATK surfactant, followed by steady further improvements in compliance over the two hour study period (Fig. 7).

In summary, the high surface and physiological activities found here for S-MB DATK synthetic surfactant are very promising for its potential future utility in treating diseases of lung surfactant deficiency and dysfunction (i.e., NRDS in preterm infants and direct pulmonary forms of ALI/ARDS in pediatric and adult patients). Synthetic lung surfactants have a number of pharmacologic advantages over animal-derived surfactants as noted earlier (greater compositional reproducibility, easier quality control, no transmission of animal disease, no animal-related ethnographic issues, and improved scale-up economy). In principle, greater chemical reproducibility and easier quality control, coupled with the removal of animal costs that have no economy of scale, should eventually translate to improved and more cost-effective surfactant therapies utilizing synthetic lung surfactant drugs.

## Supplemental Information

10.7717/peerj.1528/supp-1 Data S1 Raw data

## Additional Information and Declarations

### Data Availability

The following information was supplied regarding data availability: The raw data is available as Data S1.
